# Mutations in genes encoding antibiotic substances increase the synthesis of poly‐γ‐glutamic acid in *Bacillus amyloliquefaciens* LL3

**DOI:** 10.1002/mbo3.398

**Published:** 2016-08-18

**Authors:** Weixia Gao, Fenghong Liu, Wei Zhang, Yufen Quan, Yulei Dang, Jun Feng, Yanyan Gu, Shufang Wang, Cunjiang Song, Chao Yang

**Affiliations:** ^1^ Key Laboratory of Molecular Microbiology and Technology for Ministry of EducationNankai UniversityTianjinChina; ^2^ Key Laboratory of Bioactive Materials for Ministry of EducationState Key Laboratory of Medicinal Chemical BiologyNankai UniversityTianjinChina; ^3^ Key Laboratory of Molecular Microbiology and Technology for Ministry of EducationState Key Laboratory of Medicinal Chemical BiologyNankai UniversityTianjinChina

**Keywords:** antibiotic substance, biofilm formation, gene marker‐less deletion, poly‐γ‐glutamic acid, swarming

## Abstract

Poly‐γ‐glutamic acid (γ‐PGA) is an important natural biopolymer that is used widely in fields of foods, medicine, cosmetics, and agriculture. Several *B. amyloliquefaciens* LL3 mutants were constructed to improve γ‐PGA synthesis via single or multiple marker‐less in‐frame deletions of four gene clusters (*itu*,* bae*,* srf,* and *fen*) encoding antibiotic substances. γ‐PGA synthesis by the Δ*srf* mutant showed a slight increase (4.1 g/L) compared with that of the wild‐type strain (3.3 g/L). The Δ*itu*Δ*srf* mutant showed increased γ‐PGA yield from 3.3 to 4.5 g/L, with an increase of 36.4%. The γ‐PGA yield of the Δ*itu*Δ*srf*Δ*fen* and Δ*itu*Δ*srf*Δ*fen*Δ*bae* mutants did not show a further increase. The four gene clusters’ roles in swarming motility and biofilm formation were also studied. The Δ*srf* and Δ*bae* mutant strains were both significantly defective in swarming, indicating that bacillaene and surfactin are involved in swarming motility of *B. amyloliquefaciens* LL3. Furthermore, Δ*srf* and Δ*itu* mutant strains were obviously defective in biofilm formation; therefore, iturin and surfactin must play important roles in biofilm formation in *B. amyloliquefaciens* LL3.

## 1 Introduction

*Bacillus amyloliquefaciens* strains are ubiquitous in the soil and are great reservoirs of important natural products, such as α‐amylase, levansucrase, and fibrinolytic enzymes. Besides being powerful cell factories, *B. amyloliquefaciens* strains are also used as plant growth‐promoting and bio‐control bacteria, partly because of their ability to produce substances with antifungal, antibacterial, and nematocidal activities. The plant‐associated bacterium *B. amyloliquefaciens* FZB42, for example, has five gene clusters involved in the synthesis of lipopeptides and polyketides, which direct the synthesis of the cyclic lipopeptides surfactin, bacillomycin, fengycin, an unknown peptide, and the iron‐siderophore bacillibactin (Chen, Koumoutsi, & Scholz, 2009; Chen et al., 2009; Wu et al., 2015). Those antibiotic substances might play vital roles in *Bacillus* species living in the soil, possibly by promoting adaptation to fluctuating environmental conditions and suppressing competing bacteria or fungi (Rebecca et al., 2007; Susanne et al., 2014). However, when *B. amyloliquefaciens* cells are used as producers for desired products in the laboratory or industry, the capacity to synthesize various antibiotic substance becomes a disadvantage. It complicates the purification process and impairs the production of target products by competing for the same substrates. Those large gene clusters are also targets of genome reduction applications because they are dispensable for the cell's growth in rich media.

*B. amyloliquefaciens* LL3 is a glutamic acid‐independent poly‐γ‐glutamic acid (γ‐PGA)‐producing strain that was isolated from traditional fermented food. γ‐PGA is a promising biomaterial that is nonribosomally synthesized by the PgsBCA synthetase complex using l‐ and d‐glutamic acids as substrates, which exhibits outstanding qualities, such as good water solubility, biocompatibility, and biodegradability (Shih & Van, 2001). It is widely used in hydrogels, flocculants, drug delivery, cosmetics, and feed additives (Sung et al., 2005). *B. amyloliquefaciens* LL3 produces γ‐PGA without additional glutamic acid in the fermentation medium and thus has great potential in industrial production systems because of the lower cost and simplified process (Cao, et al., 2011). The practical use of γ‐PGA is still largely hindered by its low yield, and thus, intensive investigations have been launched to enhance its production, including optimization of fermentation conditions, modification of existing producers, and identification of new wild producers. With the availability of more and more gene manipulation methods, genome‐scale modification of existing producers becomes affordable.

In the past decades, strategies to improve γ‐PGA production were limited to optimization of medium and fermentation conditions. In the 21st century, there have been some attempts to improve the γ‐PGA yield using metabolic engineering strategies. Yeh, Wang, Lo, Chan, and Lin (2010) integrated an efficient synthetic expression control sequence (SECS) into the upstream region of the silent *pgsBCA* gene cluster of *B. subtilis* DB430 to produce γ‐PGA. Liu et al. (2011) enhanced γ‐PGA productivity by depressing exopolysaccharides production. VHb (*Vitreoscilla* hemoglobin) alleviates the oxygen limitation at the later stage of fermentation. The encoding gene, *vgb*, has been successfully expressed in a γ‐PGA‐producing strain to improve γ‐PGA production, especially under oxygen‐limited conditions (Richard & Margaritis, 2003; Zhang et al. 2013; Su et al., 2010). Heterologous expression of the γ‐PGA synthetase complex (*pgsBCA*) is another strategy for γ‐PGA production improvement, which has been carried out in coryneform bacteria. *Corynebacterium glutamicum* E12 harboring vector pMT‐HCE‐*pgsBCA* could express γ‐PGA synthetase genes from *B. subtilis* and could be considered as a host for γ‐PGA synthesis (Sung et al., 2005). Feng et al. (2015) and Feng, Gu, Sun, Han, Yang et al. (2014) enhanced γ‐PGA production of *B. amyloliquefaciens* LL3 by metabolically engineering its γ‐PGA synthesis‐related metabolic networks: by‐products synthesis, γ‐PGA degradation, glutamate precursor synthesis, γ‐PGA synthesis, and autoinducer synthesis. However, few reports have focused on the antibiotic substances, which may compete with γ‐PGA for similar synthesis machinery or substrates.

The whole genome of *B. amyloliquefaciens* LL3 was sequenced (Geng et al. 2011), and several gene clusters responsible for the synthesis of antibiotic substances were found, including the *bae*,* srf*,* fen,* and *itu* clusters, as shown in Figure S1. The *bae* cluster (annotated as *pks* in *B. subtilis* 168) encodes bacillaene, which was originally discovered as a bacteriostatic agent that inhibited prokaryotic protein synthesis. Surfactin, iturin A, and fengycin, encoded by the *srf*,* itu,* and *fen* clusters, respectively, are nonribosomally produced cyclic lipopeptides that act against phytopathogenic viruses, bacteria, fungi, and nematodes.

**Figure 1:**
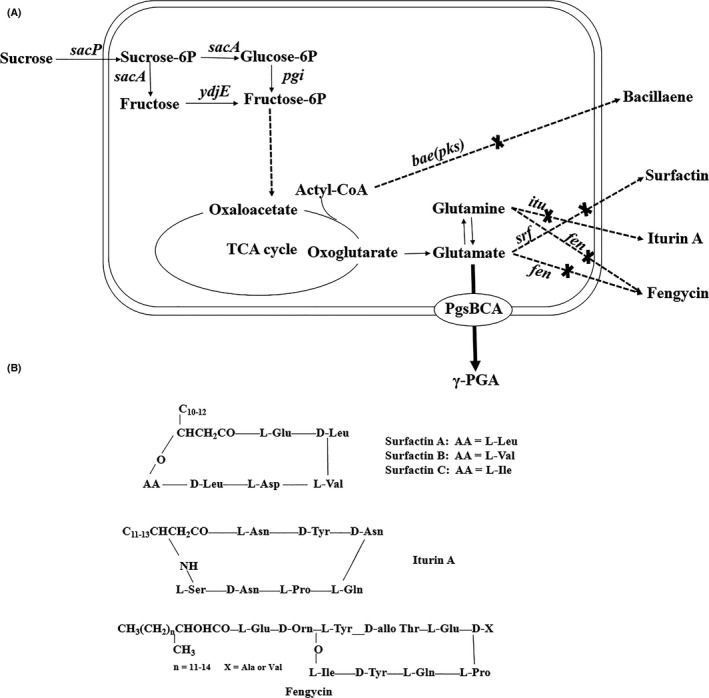
(A) Schematic of modular engineering approach in *Bacillus amyloliquefaciens* LL3 strain. The X marks indicate the gene deletions in the optimized pathway. (B) Condensed structural formulae of the three cyclic lipopeptides, surfactin, iturin A, and fengycin, produced by *B. amyloliquefaciens* LL3

A transcriptional comparison between *B. amyloliquefaciens* LL3 (γ‐PGA^+^) and LL3Δ*pgsBCA* (γ‐PGA^−^) was performed using RNA‐*seq* (unpublished data). Interestingly, the transcript levels of the *bae*,* srf*,* itu,* and *fen* clusters experienced a sharp increase in *B. amyloliquefaciens* LL3 Δ*pgsBCA* (Table 1). Specifically, the expression levels of the first genes of the four aforementioned clusters in *B. amyloliquefaciens* LL3Δ*pgsBCA* were 14.42‐, 8.08‐, 12‐, and 9.93‐fold higher than that in *B. amyloliquefaciens* LL3. This suggested that the synthesis of γ‐PGA may suppress the transcription of the above four clusters. Hence, we proposed that the synthesis of the four antibiotic substances may consequently suppress the γ‐PGA synthesis directly or indirectly in turn. In addition, bacillaene, surfactin, iturin A, and fengycin as well as γ‐PGA are all nonribosomally produced. Moreover, surfactin, iturin A, and fengycin contain several glutamates or glutamines, which are the precursors of γ‐PGA (Fig. 1B). Besides, acetyl‐CoA, main precursor of bacillaene, plays important role in TCA cycle, which offers glutamate as precursor for γ‐PGA synthesis. What is more, lipopeptides or polyketides may be viewed as costly for the cells from a metabolic point of view given the big size of the corresponding operons. Therefore, those four antibiotic substances may share similar synthesis machinery and compete for common substrates with γ‐PGA. Based on these speculations, we attempted to obtain a γ‐PGA producer with higher yield and purity, using single or combined marker‐less deletions of the *itu*,* bae*,* srf,* and *fen* gene clusters (Fig. 1A). Their roles in swarming and biofilm formation were also investigated.

**Table 1:** Comparison of expression level of the genes which encode the four antibiotic substance between the *B. amyloliquefaciens* LL3 (γ‐PGA^+^) and LL3 Δ*pgsBCA* (γ‐PGA^−^)

Gene	Length	Product	Foldchange
*srfA*	10755	Nonribosomal surfactin synthetase, SrfAA	8.08
*srfB*	10761	Nonribosomal surfactin synthetase, SrfAB	12.37
*srfC*	3840	Nonribosomal surfactin synthetase C, SrfC	12.51
*srfD*	732	Nonribosomal surfactin synthetase D, SrfD	10.46
*sfp*	675	Surfactin synthetase‐activating enzyme	1.69
*ituC*	7851	Iturin A synthetase C, ItuC	9.32
*ituB*	16086	Iturin A synthetase B, ItuB	10.52
*ituA*	11949	Iturin A synthetase A, ItuA	12.02
*ituD*	1203	Malonyl‐CoA transacylase, ItuD	1.86
*fenE*	3846	Fengycin synthetase E, FenE	11.43
*fenD*	7677	Fengycin synthetase D, FenD	9.93
*baeB*	693	Polyketide biosynthesis zinc‐dependent hydrolase, BaeB	1.07
*baeC*	870	Polyketide biosynthesis malonyl‐CoA‐acyl‐carrier‐protein transacylase, BaeC	1.94
*baeD*	975	Polyketide biosynthesis acyltransferase homolog, BaeD	3.23
*baeE*	2238	Polyketide biosynthesis protein, BaeE	4.14
*acpK*	249	Polyketide biosynthesis acyl‐carrier‐protein, AcpK	1.63
*baeG*	1263	Polyketide biosynthesis 3‐hydroxy‐3‐methylglutaryl‐ACP synthase, PksG	6.50
*baeH*	774	Probable polyketide biosynthesis enoyl‐CoA hydratase, PksH	6.17
*baeI*	750	Putative polyketide biosynthesis enoyl‐CoA isomerase, PksI	4.95
*baeJ*	14952	Polyketide synthase, PksJ	11.78
*baeL*	13431	Polyketide synthase, PksL	15.59
*baeL*	10542	Polyketide synthase, PksM	11.12
*baeM*	16314	Polyketide synthase, PksN	12.64
*baeR*	7446	Polyketide synthase, PksR	14.42
*baeS*	1212	Polyketide biosynthesis cytochrome P450, PksS	2.11

## 2 Experimental Procedures

### 2.1 Strains, media, and culture conditions

*E. coli* DH5α was used for plasmid construction. *E. coli* GM2163 was used to prepare the unmethylated plasmids for subsequent use in the electroporation of *B. amyloliquefaciens* strains. *E. coli* strains were cultured in LB medium at 37°C with aeration. The *B. amyloliquefaciens* LL3 strain was deposited in the China Center for Type Culture Collection (CCTCC) with accession number CCTCC M 208109. *B. amyloliquefaciens* LL3 and its derivatives were cultured at 37°C in LB or fermentation medium for growth and γ‐PGA synthesis experiments, at 30°C when the temperature‐sensitive deletion plasmid was introduced, or at 42°C when plasmid integration/excision was performed during gene deletion. Fermentation medium for *B. amyloliquefaciens* LL3 and its derivatives contained sucrose 50 g/L, (NH_4_)_2_SO_4_ 2 g/L, MgSO_4_ 0.6 g/L, KH_2_PO_4_ 6 g/L, K_2_HPO_4_ 14 g/L, 2 mL mineral elements including 1 mmol/L FeSO_4_•4H_2_O, CaCl_2_•2H_2_O, MnSO_4_•4H_2_O, and ZnCl_2_ (pH 7.2). When required, media were supplemented with ampicillin (Ap; 100 μg/mL), chloramphenicol (Cm; 5 μg/mL), or tetracycline (Tc; 10 μg/mL).

### 2.2 Plasmid construction and bacterial transformation

The plasmids and primers used in this study are listed in Table 2 and Table 3. Temperature‐sensitive plasmid pKSV7 is a shuttle vector for *E. coli* and *Bacillus*, which is stable at 30°C or below and unstable at 37°C or above. Counter‐selective plasmid pKSU is a derivative of pKSV7 that carries the *upp* gene from *B. subtilis* 168. Sequences up‐ and downstream of the target gene clusters were PCR amplified and spliced in a subsequent overlapping PCR. The resulting homologous arms were digested with *Bam*HI and *Sal*I and ligated in the same restriction sites of pKSU to yield deletion plasmids. DNA polymerases, restriction enzymes, and T4 DNA ligase were purchased from Takara (Dalian, China). PCR, enzyme digestion, and ligation reactions were performed as recommended by the enzyme suppliers. The DNA fragments were analyzed on 0.8% agarose gels and purified using an Axygen gel DNA recovery kit (Axygen, CA, USA). Deletion plasmids were treated with *Bam* HI methyltransferase (New England Biolabs, MA, USA) before transformed into *B. amyloliquefaciens* strains.

**Table 2:** Strains and plasmids used in this study

Plasmids or Strains	Description	Source
Plasmids		
pKSU	pKSV7 carrying the *upp* gene from *B. subtilis* 168, used for counterselection	Zhang et al. (2014)
pKSU‐Δ*srf*	pKSU carrying a mutant copy of the *srf* cluster	This study
pKSU‐Δ*itu*	pKSU carrying a mutant copy of the *itu* cluster	This study
pKSU‐Δ*fen*	pKSU carrying a mutant copy of the *fen* cluster	This study
Strains		
*B. amyloliquefaciens*		
LL3	Glutamic acid‐independent poly‐γ‐glutamic acid (γ‐PGA)‐producing strain	Geng et al. (2011)
LL3Δ*upp*	LL3 carrying an in‐frame deletion in the *upp* gene	Zhang et al. (2014)
LL3Δ*pgsBCA*	LL3 Δ*upp* deleted for *pgsBCA*	Unpublished
LL3Δ*bae*	LL3 Δ*upp* deleted for its partial *bae* cluster	This study
LL3Δ*srf*	LL3 Δ*upp* deleted for the *srf* cluster	This study
LL3Δ*itu*	LL3 Δ*upp* deleted for the *itu* cluster	This study
LL3Δ*fen*	LL3 Δ*upp* deleted for the *fen* cluster	This study
LL‐IS	LL3Δ*upp*Δ*itu* deleted for the *srf* cluster	This study
LL‐ISF	LL‐IS deleted for the *fen* cluster	This study
LL‐ ISFB	LL‐ISF deleted for the *bae* cluster	This study
*E. coli* strains		
DH5α	*supE44* Δ*lacU169*(*_80 lac*ZΔM15) *recA1 endA1 hsdR17*(rK^−^ mK^+^) *thi*‐*1gyrA relA1 *F^−^ Δ(*lacZYA*‐*argF*)	TransGen
GM2163	F^−^ *dam‐13::*Tn*9* (Cam^r^) *dcm‐6 hsdR2* (r_k_^−^m_k_^+^) *leuB6 hisG4 thi‐1 araC14 lacY1 galK2 galT22 xylA5 mtl‐1 rpsL136* (Str^r^) *fhuA31 tsx‐78 glnV44 mcrA mcrB1*	Fermentas

**Table 3:** Oligonucleotide primers used in this study

Primer names	Sequence (5′–3′)^a^
BaeUP‐F	CGG**TCTAGA**AAACTACATGTCATCTGTCATTAACG
BaeUP‐R	CATCGAGAAGTTCTTAAAAGATCCGGGCAGAC
BaeDN‐F	CCCGGATCTTTTAAGAACTTCTCGATGCCTAC
BaeDN‐R	TGA**GTCGAC**GTGACGGCTTCTCTTTCAG
BaeOUT‐F	ATGATACCGCTCCATGTCAGCTCACTTG
BaeOUT‐R	CGCCGTGCTTCGTTCATCTAATTCG
SrfUP‐F	GCC**GTCGAC**ATGGGAATAACTTTTTATCC
SrfUP‐R	GGCATCGATATTGCTCCAGAGATACTGTAAAC
SrfDN‐F	CAGTATCTCTGGAGCAATATCGATGCCGATCG
SrfDN‐R	CGC**GGATCC**ATCTTTAACCATTAAAGGAAAAG
SrfOUT‐F	GGAGGCTGTTTCTAAGGAAGAATTGAC
SrfOUT‐R	GACGTTTTATTTTGCCGGTCTGTTG
FenUP‐F	TGT**GGATC**CCTATCTTGCCCTCTGTCTTC
FenUP‐R	AGAAATATCCTTACGCAAACGGCAAAGTGGACC
FenDN‐F	TTTGCCGTTTGCGTAAGGATATTTCTGGTGCCG
FenDN‐R	GCA**GTCGAC**TTGAAGAATACTGTTTATGCTT
FenOUT‐F	AATGGGTCAGCCGGTAGCTGGCAAG
FenOUT‐R	TGCGTCAAATTCAGGGGAAACATCG
ItuUP‐F	CGA**GGATCC**AAATTGAGGCAATAGGAATAG
ItuUP‐R	TAACAGTCAGTGTGTTGGGATCGTTTGCGGGAGAC
ItuDN‐F	GCAAACGATCCCAACACACTGACTGTTAAAATAGC
ItuDN‐R	CGA**GTCGAC**TGGGGGCTTCACAATGATTTATGT
ItuOUT‐F	CGGTCATGTAGCCGATCTCACCTGG
ItuOUT‐R	ATTGAAATCTTCCGAATGGTGCTTG
qRpsU‐F	GTCGTTAGAAAAAACGAATCGCTTG
qRpsU‐R	TTGCGTTTTCTAGCAGCTTCTGACT
qPgsB‐F	TAGCCTGTGCTGCCGTACTAATCAT
qPgsB‐R	GTTTCCGTTTGATCGGTTTCTCCT

a Restriction sites used for the cloning of PCR amplicons are indicated in bold

Competent *E. coli* cells were purchased from Transgen Biotech (Beijing, China) and transformed according to the manufacturer's instructions. Deletion plasmids were transformed into *B. amyloliquefaciens* strains using the high osmolarity electroporation method, with modifications, as described previously (Zhang et al., 2014).

### 2.3 Markerless deletion of the four gene clusters

Gene deletions in this study were carried out adapting a previously reported markerless gene replacement method based on *upp* and will be described briefly below (Zhang et al., 2014). *B. amyloliquefaciens* LL3Δ*upp* carrying an in‐frame deletion of *upp* and that is resistant to 1.3 mmol/L 5‐fluorouracil (5‐FU) was used as the parental strain for subsequent mutants construction. Introduction of the deletion plasmid pKSU would restore sensitivity to 5‐FU for *B. amyloliquefaciens* LL3 Δ*upp* and its derivatives.

Deletion of the *srf* cluster will be used as an example to explain the method. The up‐ and downstream homologous arms (~1 kb each) used for *srf* deletion were obtained using primer pairs SrfUP‐F/SrfUP‐R and SrfDN‐F/SrfDN‐R, respectively. These two fragments were then spliced in a subsequent overlapping PCR using primer pair SrfUP‐F/SrfDN‐R. The resultant homologous arms were ligated into pKSU to yield the deletion plasmid pKSU‐Δ*srf*, which was then transformed into *B. amyloliquefaciens* LL3 Δ*upp* in the presence of Cm at 30°C. After plasmid establishment, the recombinants were cultured at 42°C in the presence of Cm to facilitate chromosomal integration. The obtained single‐crossover recombinants were then grown in LB in the absence of Cm to facilitate plasmid excision from the genome. Cultures were diluted and plated in LB agar supplemented with 5‐FU. Deletion‐carrying strains were designated *B. amyloliquefaciens* LL3Δ*srf* and were confirmed by PCR, using primer pair SrfOUT‐F/SrfOUT‐R, and DNA sequencing. Mutants deleted for *bae*,* itu*, and *fen* gene clusters were similarly constructed and were designated *B. amyloliquefaciens* LL3Δ*bae*, LL3Δ*itu*, and LL3Δ*fen*, respectively.

*B. amyloliquefaciens* LL3Δ*itu* was transformed with pKSU‐Δ*srf* to obtain LL‐UIS (Δ*srf*Δ*itu*), which carries double deletions of the *itu* and *srf* clusters. The *fen* cluster was then deleted in strain LL‐IS to obtain LL‐ISF (Δ*srf*Δ*itu*Δ*fen*), which carries triple deletions. Finally, the *bae* cluster was accumulated in strain LL‐UISF to yield LL‐ISFB (Δ*srf*Δ*itu*Δ*fen*Δ*bae*), which is deficient in all four antibiotic substances.

### 2.4 Cell growth and γ‐PGA synthesis

Fresh colonies of *B. amyloliquefaciens* strains were first grown overnight in test tubes containing 5 mL LB liquid and then inoculated into 100 mL fresh LB or fermentation medium in 500‐mL shake flasks to an optical density of approximately 0.05–0.1, at 600 nm (OD_600_). The shake flasks were then incubated at 37°C for 48 h with an agitation at 200 rpm. For growth experiments, 1‐mL culture was withdrawn periodically to determine the OD_600_. At the end of fermentation, the viscosity of the culture was determined using a viscosimeter (Brookfield DV‐I+, MA, USA). For dry cell weight (DCW) and γ‐PGA synthesis determination, 100 mL cultures were centrifuged at 8,000*g* (4°C) for 20 min. The cell pellet was washed three times with dH_2_O and then dried and weighed to determine the DCW. The supernatant was used to extract γ‐PGA, using an ethanol precipitation method, as previously described (Zhang et al., 2013). Experiments were independently repeated at least three times, and the means and standard deviations were calculated.

### 2.5 qRT‐PCR analysis of the *pgsB* gene

The wild‐type *B. amyloliquefaciens* LL3 and its derivatives were grown to mid‐log phase (approximately 20 hr) in fermentation medium. The cells were collected at 4°C and RNA was isolated using *TransZolTM* Up (TransGen, Beijing, China), according to the manufacturer's instructions. cDNA was reverse transcribed using a GoScriptTM Reverse Transcription System (Promega, WI, USA). Real‐time PCR analysis for the target genes was performed using the SYBR^®^PremixEx *Taq* ^*TM*^ II (Takara, Dalian, China). Transcript levels of the target genes were normalized against the levels of *rspU* (Feng, Goa, Gu, Zang, Cao et al., 2014).

### 2.6 Isolation of cyclic lipopeptides and HPLC‐MS analysis

Isolation of surfactins, fengycins, and iturin A and HPLC‐MS analysis were carried out using a method described previously (Luo, Liu, Zhou, Wang, & Chen, 2015). All the samples were further analyzed by matrix‐assisted laser desorption ionization‐time of flight mass spectrometry (MS) with a Shimadzu 2020 series HPLC‐MS/MS system (Shimadzu, Japan).

### 2.7 Swarming and biofilm formation experiments

Fresh colonies of strains to be tested were inoculated and cultivated overnight in LB medium. Then, 10‐μL culture was spotted in the middle of plates containing different agarose concentrations (0.25%, 0.5%, and 0.7%) and incubated for 24 hr.

The biofilm formation experiment was performed as described by Feng, Gu et al. (2014). Overnight cultures of the wild‐type and its derivatives were diluted to an OD_600_ of 1.0 in fresh LB medium. Samples of 10 μl of the diluted cells were then added to 10 ml of MSgg broth in six‐well microtiter dish. The dish was incubated for 72 hr at 30°C without stir.

## 3 Results

### 3.1 Construction of recombinant strains carrying single‐ or multiple‐gene deletions

**Figure 2:**
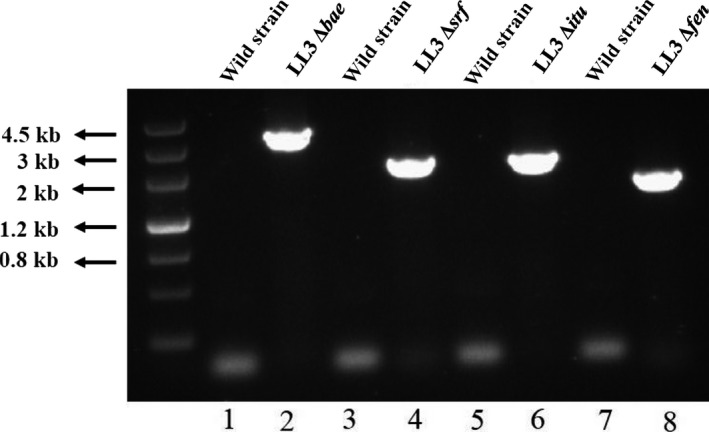
Confirmation of the deletion of the genes by agarose gel electrophoresis of PCR products with primers BaeOUT‐F/R (lanes 1 and 2), SrfOUT‐F/R (lanes 3 and 4), ItuOUT‐F/R (lanes 5 and 6), and FenOUT‐F/R (lanes 7 and 8). Chromosomal DNA of the mutant strains served as the template for PCR. Fragments of the wild‐type strain were too long to obtain PCR products

The marker‐less gene knockout method was used to construct the gene deletion mutants, using the *upp* cassette and 5‐fluorouracil (5‐FU) selection (Zhang et al., 2014). The primers BaeOUT‐F/R, SrfOUT‐F/R, ItuOUT‐F/R, and FenOUT‐F/R were used to confirm the construction of gene deletion mutants. As shown in Figure 2, the single, double, triple, and quadruple mutants of the *bae*,* srf*,* itu,* and *fen* genes were successfully generated, and the single‐gene deletion mutants were designated as *B. amyloliquefaciens* LL3Δ*bae*, LL3Δ*srf*, LL3Δ*itu,* and LL3Δ*fen*, respectively. The multiple‐gene deletion mutants were named LL‐IS (Δ*srf*Δ*itu*), LL‐ISF (Δ*srf*Δ*itu*Δ*fen*), and LL‐ISFB (Δ*srf*Δ*itu*Δ*fen*Δ*bae*), respectively.

### 3.2 Swarming ability and biofilm formation of the mutant strains

**Figure 3:**
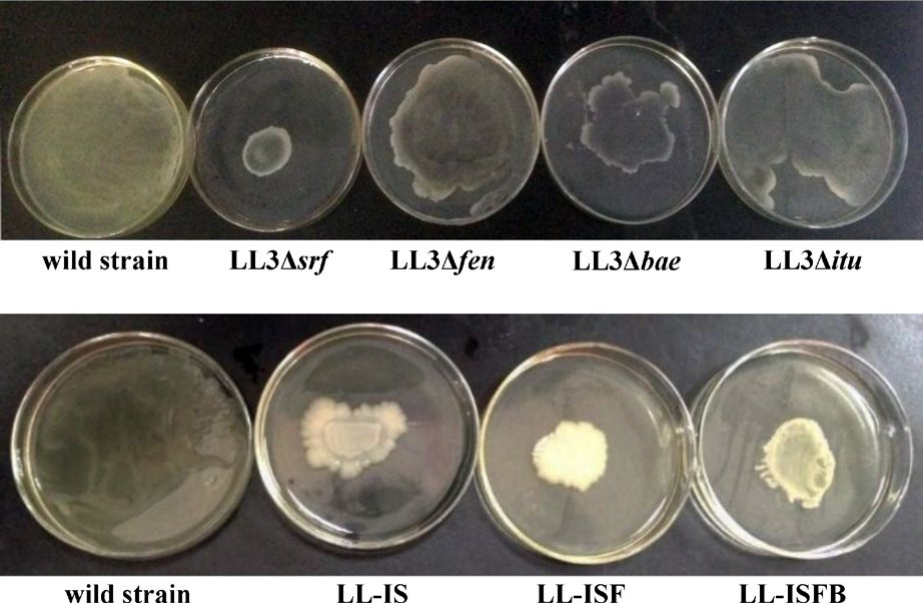
Swarming experiments of the wild‐type strain, *B. amyloliquefaciens* LL3Δ*srf*, LL3Δ*fen*, LL3Δ*bae*, LL3Δ*itu*, LL‐IS (Δ*itu*Δ*srf*), LL‐ISF (Δ*itu*Δ*srf*Δ*fen*), and LL‐ISFB (Δ*itu*Δ*srf*Δ*fen*Δ*bae*). Strains were observed after 24‐hr cultivation on LB medium with 0.5% agar

Swarming is a social motility behavior found in *Bacillus* strains and is associated with biofilm development. As previously reported, surfactin plays important roles in the swarming of *B. subtilis* strains (Kearns et al. 2004). In the “swim plates” (LB plate with 0.25% agar), all the mutants generated colonies that spread over the plate and showed efficient swimming motility (data not shown). However, as shown in Figure 3, B*. amyloliquefaciens* LL3Δ*srf* and LL3Δ*bae* showed obvious defects in swarming motility in the “swarm plates” (LB plate with 0.5% agar), while *B. amyloliquefaciens* LL3Δ*fen* and LL3Δ*itu* showed slight defects in swarming. This agreed with the previous reports that the *srf* gene cluster is involved in swarming motility.

All the multiple‐gene mutants, *B. amyloliquefaciens* LL‐IS (Δ*itu*Δ*srf*), LL‐ISF (Δ*itu*Δ*srf*Δ*fen*), and LL‐ISFB (Δ*itu*Δ*srf*Δ*fen*Δ*bae*), had significant defects in swarming motility (Fig. 3). LL3Δ*itu* had a slight defect in swarming; however, when *srf* was deleted to construct LL‐IS (Δ*itu*Δ*srf*), its swarming motility was significantly weakened, which further proved surfactin's effect on swarming.

**Figure 4:**
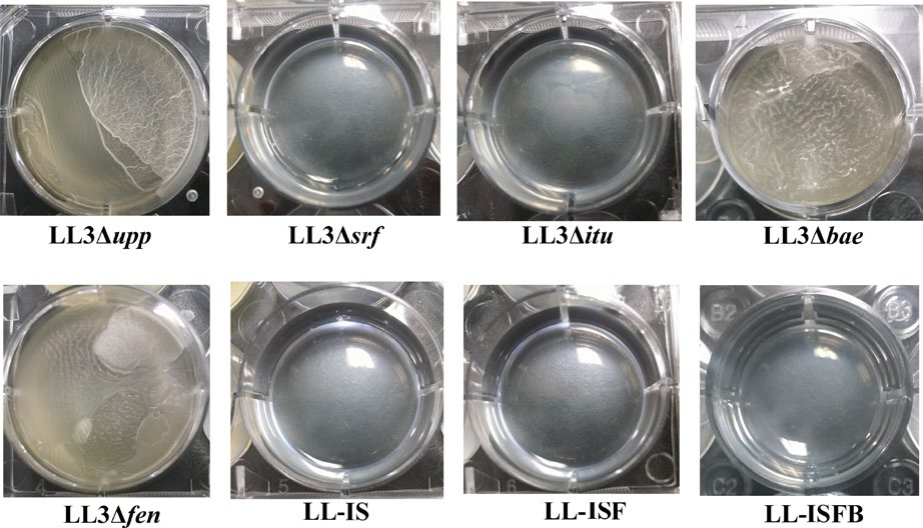
Biofilm formation of the wild‐type strain, *B. amyloliquefaciens* LL3Δ*srf*, LL3Δ*itu*, LL3Δ*bae*, LL3Δ*fen*, LL‐IS (Δ*itu*Δ*srf*), LL‐ISF (Δ*itu*Δ*srf*Δ*fen*), and LL‐ISFB (Δ*itu*Δ*srf*Δ*fen*Δ*bae*)

Rahman et al. (2007)reported that the biofilm formation of transformant *B. subtilis* RM/iSd16 containing wild *sfp*,* itu* operon, and *degQ* was better than the wild‐type strain. Zeriouh, de Vicente, Perez‐Garcia, and Romero (2014) found that surfactin triggered biofilm formation of *B. subtilis*. In this study, the biofilm formation ability of all the mutant strains was compared with that of the wild‐type strain. *B. amyloliquefaciens* LL3Δ*bae* and LL3Δ*fen* showed similar biofilm formation compared with the wild‐type strain (Fig. 4). *B. amyloliquefaciens* LL3Δ*srf*, LL3Δ*itu*, LL‐IS, LL‐ISF, and LL‐ISFB were significantly defective in biofilm formation (Fig. 4). This could be inferred that iturin A and surfactin play important roles in biofilm formation.

### 3.3 DCW, γ‐PGA synthesis and culture viscosity of the mutant strains

**Figure 5:**
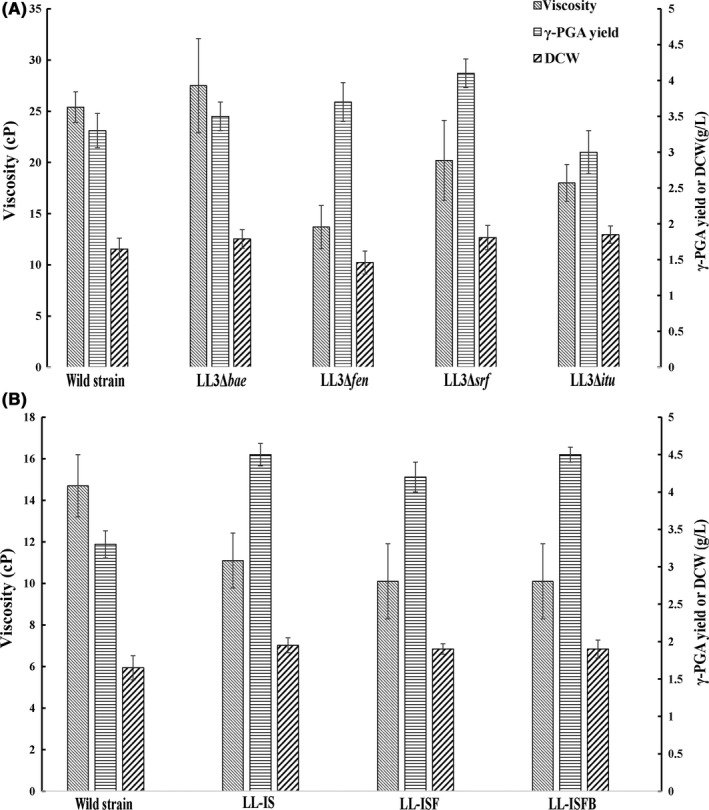
(A) Comparison of culture viscosity, DCW, and γ‐PGA yield of the wild‐type strain and the mutant strains carrying single‐gene deletion (*B. amyloliquefaciens* LL3Δ*srf*, LL3Δ*bae*, LL3Δ*fen,* and LL3Δ*itu*) after 48 hr of cultivation; (B) Comparison of culture viscosity, DCW, and γ‐PGA yield of the wild‐type and mutant strains carrying multiple deletions (*B. amyloliquefaciens* LL‐IS, LL‐ISF, and LL‐ISFB) after 48 hr of cultivation

*B. amyloliquefaciens* LL3Δ*bae*, LL3Δ*srf*, LL3Δ*itu,* LL3Δ*fen*, LL‐IS (Δ*itu*Δ*srf*), LL‐ISF (Δ*itu*Δ*srf*Δ*fen*), and LL‐ISFB (Δ*itu*Δ*srf*Δ*fen*Δ*bae*) were compared with the wild‐type strain for culture viscosity, DCW, and γ‐PGA synthesis. At the end of the fermentation, surprisingly, the culture viscosity of the Δ*srf*, Δ*itu,* and Δ*fen* mutants was decreased by 46%, 20.5%, and 29%, respectively, while the Δ*bae* mutant showed no apparent changes compared with the wild‐type strain (Fig. 5A). The DCW of the Δ*fen* mutant experienced a slight decrease, while that of the other three mutants resembled the wild‐type strain. γ‐PGA synthesis of the Δ*fen*, Δ*itu,* or Δ*bae* mutants showed no obvious changes; however, in the Δ*srf* mutant, the synthesis of γ‐PGA showed a slight increase (4.1 g/L) compared with that in the wild‐type strain (3.3 g/L).

As shown in Figure 5B, culture viscosities of LL‐IS (Δ*itu*Δ*srf*), LL‐ISF (Δ*itu*Δ*srf*Δ*fen*), and LL‐ISFB (Δ*bae*Δ*srf*Δ*itu*Δ*fen*) were decreased by 24.6%, 31%, and 31%, respectively. The DCW of LL‐IS (Δ*itu*Δ*srf*), LL‐ISF (Δ*itu*Δ*srf*Δ*fen*), and LL‐ISFB (Δ*bae*Δ*srf*Δ*itu*Δ*fen*) were increased by 18%, 15%, and 15%, respectively. The γ‐PGA synthesis of LL‐IS (Δ*itu*Δ*srf*) increased by 36.4%, leading to a yield of 4.5 g/L, compared with 3.3 g/L in the wild‐type control, while LL‐ISF(Δ*itu*Δ*srf*Δ*fen*) showed a slight decrease compared with LL‐IS (Δ*itu*Δ*srf*) and the γ‐PGA yield of LL‐ISFB (Δ*bae*Δ*srf*Δ*itu*Δ*fen*) resembled that of LL‐IS (Δ*itu*Δ*srf*).

## 4 Discussion

γ‐PGA‐producing strains are generally divided into two groups according to their nutritional requirements: glutamic acid‐dependent bacteria and glutamic acid‐independent bacteria. The latter does not require additional l‐glutamate in the medium to stimulate γ‐PGA, so that their production costs are lower than the former.

*B. amyloliquefaciens* LL3 is a naturally isolated, Gram‐positive strain that can produce γ‐PGA without the addition of glutamic acid in the medium. It secretes various antibiotic substances to adapt to the environment, such as surfactin, iturin A, fengycin, and bacillaene. Except for bacillaene, all of them are lipopeptides. Many reports have shown that the biological control exerted by *B. subtilis* and related species could be attributed to nonribosomally produced cyclic lipopeptides (Ongena & Jacques, 2008; Pérez‐García, Romero, & de Vicente, 2011; Romero, de Vicente, Rakotoaly, Dufour, Veeing, Arrebola, 2007; Zeriouh et al., 2011). Lipopeptides interact with the biological membranes of microbial pathogens, inducing cell leakage and death (Romero, de Vicente, Olmos, Davila, & Pérez‐García, 2007; Zeriouh et al., 2011).

In *B. amyloliquefaciens* LL3, the gene cluster encoding surfactin consists of *srfA*,* srfB*,* srfC*,* srfD,* and *sfp*, which encode surfactin synthetase A, B, C, D and surfactin kinase, respectively. The iturin A‐encoding cluster contains *ituA*,* ituB*,* ituC,* and *ituD*. The fengycin‐encoding gene cluster comprises *fenD* and *fenE*. Bacillaene is a polyketone, which is encoded by a gene cluster comprising *baeB*,* baeC*,* baeD*,* baeE*,* baeG*,* baeH*,* baeI*,* baeJ*,* baeL*,* baeM*,* baeR,* and *baeS*. The total lengths of the four gene clusters are 28.3, 37.2, 11.5, and 72.5 kb, respectively (Fig. S1). They were all predicted as nonessential using a comparative genomics approach and comparing the *B. amyloliquefaciens* LL3 genome with the *B. subtilis* 168 genome (Database of Essential Genes, DEG, http://tubic.tju.edu.cn/deg/). The four antibiotic substances are all nonribosomally produced, like γ‐PGA. Therefore, they may share similar synthesis machinery and compete for substrates with γ‐PGA. Interestingly, a transcriptional comparison between *B. amyloliquefaciens* LL3 (γ‐PGA^+^) and LL3 Δ*pgsBCA* (γ‐PGA^−^) using RNA‐seq agreed with the above speculation (unpublished). Transcriptional levels of *bae*,* srf*,* itu,* and *fen* clusters are greatly enhanced in *B. amyloliquefaciens* LL3 Δ*pgsBCA*. Based on these results, it was decided to knock out the four gene clusters to improve γ‐PGA yield and purity.

Biofilm formation and swarming are typical characteristics of the *Bacillus* genus. However, few reports took *B. amyloliquefaciens* as object of study. At first, we detected the effects of antibiotic substances encoding gene clusters disruption on the biofilm formation and swarming ability in *B. amyloliquefaciens*. Ghelardi et al. (2012) showed that both SwrA and surfactin upregulate the transcription of the flagellin gene and increase bacterial swimming ability in *B. subtilis*. In this study, *B. amyloliquefaciens* LL3Δ*srf* and LL3Δ*bae* showed obvious defects in swarming motility in the “swarm plates,” which correlates with previous reports that the *srf* gene cluster is involved in swarming motility (Kearns et al. 2004). Besides, LL3Δ*srf* and LL3Δ*itu* were significantly defective in biofilm formation. This could be inferred that iturin A and surfactin play important roles in biofilm formation, which was in line with the previous study (Ghelardi et al., 2012; Luo et al. 2015).

Principally, we focused on the effects of gene clusters disruption on γ‐PGA production. Results showed that *B. amyloliquefaciens* LL3Δ*srf* was the only single‐deletion mutant strain that showed an increase in γ‐PGA production (by 24.2%) compared with the wild‐type strain. Many reports have discovered that the presence of Mn^2+^, a cofactor for glutamine synthetase (GS), in the medium can improve the yield of surfactin during fermentation of *B. subtilis* (Abdel‐Mawgoud, Aboulwafa, & Hassouna, 2008; Huang, Liu, Wang, Liu, & Lu, 2015). Glutamine synthetase is an enzyme that catalyzes L‐glutamate to glutamine and plays important roles in glutamate consumption. This is good evidence that surfactin shares same substrates with γ‐PGA. Besides, as mentioned above, surfactin upregulates the transcription of the flagellin gene (Ghelardi et al., 2012). Chan, Guttenplan, and Kearns (2014) found that defects in the flagellar motor increase synthesis of poly‐γ‐Glutamate in *B*.* subtilis*. Therefore, *srf* mutant may also enhance γ‐PGA synthesis indirectly.

**Figure 6:**
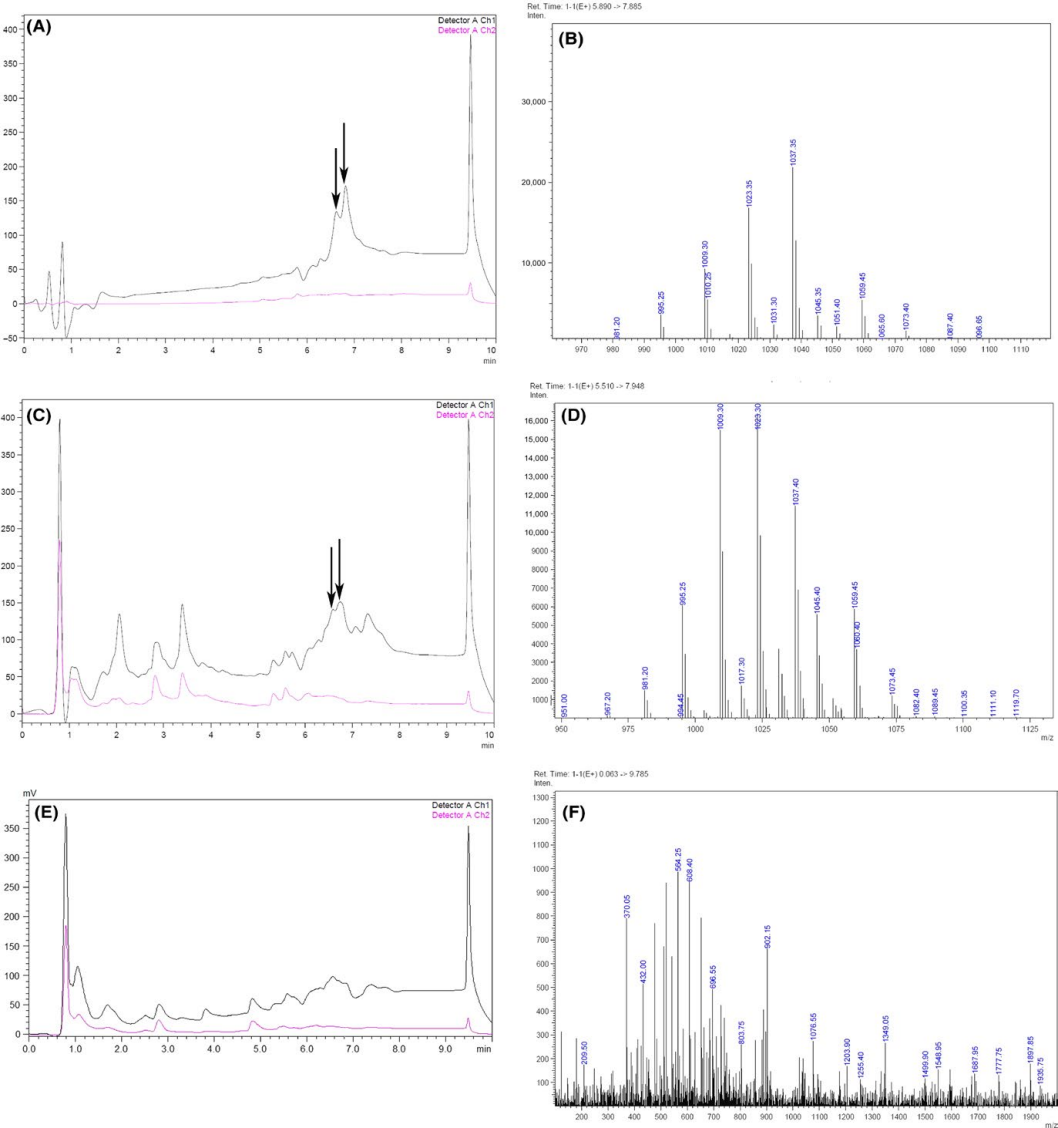
HPLC‐MS spectrograms of standard surfactin and surfactin produced by the wild strain and LL3Δ*srf*. (A, B) HPLC and MS spectrograms of standard surfactin. (C, D) HPLC and MS spectrograms of surfactin from the wild strain. (E, F) HPLC and MS spectrograms of the LL3Δ*srf* culture broth, which was disrupted in *srf* cluster and deficient in production of surfactin

However, *B. amyloliquefaciens* LL3Δ*bae*, LL3Δ*fen,* and LL3Δ*itu* did not show significant increases in γ‐PGA yield. For further explanation, HPLC‐MS was used to detect whether all the strains could produce the antibiotic substances or not. As shown in Figure 6, B*. amyloliquefaciens* LL3Δ*upp* could synthesize surfactin, while LL3Δ*srf* cannot produce surfactin anymore. It may be the main reason for the increase of γ‐PGA synthesis that surfactin competes for same substrates with γ‐PGA. In addition, iturin A and fengycin were undiscovered in the culture of LL3Δ*upp* (Fig. S3). This can explain why LL3Δ*itu* and LL3Δ*fen* showed no increases in γ‐PGA synthesis.

It was further examined whether the disruption of the four gene clusters affected the expression of *pgs* operon. As shown in Figure S2, the *pgsB* expression levels of these mutant strains were comparable; thus, it can be presumed that the γ‐PGA synthesis changes in mutant strains are not likely related to the *pgs* operon expression level.

LL‐IS showed slight increase in γ‐PGA titer compared with LL3Δ*srf* although we did not discover iturin A in the culture of the wild strain. However, as mentioned above, LL3Δ*itu* was significantly defective in biofilm formation. It is speculated that LL3Δ*upp* might synthesize other iturin derivatives, which may also contributes to biofilm formation. Accumulation of multiple‐gene cluster deletions in one strain, LL‐ISF and LL‐ISFB did not give rise to a continuous increase in γ‐PGA yield. This may be attributed to that the wild strain might not produce fengycin or bacillaene and that the secondary metabolites might act not only as antibiotic substances but also as signal molecules affecting various cellular activities.

We previously reported that *Vitreoscilla* hemoglobin (VHb) alleviated the oxygen limitation leading to increased γ‐PGA production. Being too viscous to stir is an important factor of oxygen limitation during γ‐PGA fermentation. So we detected the culture viscosities of all the strains and found that culture viscosities of LL‐IS, LL‐ISF, and LL‐ISFB decreased by 24.6%, 31%, and 31%, respectively, compared with the wild‐type strain. This may be related to surfactin and iturin playing important roles in biofilm formation. Lower viscosity is very beneficial for industrial production.

Unfortunately, the four gene clusters are very large such that it would be difficult to construct the corresponding complementary strains to confirm the four products’ roles in cell motility, biofilm formation, and γ‐PGA synthesis, although our results are mainly in accordance with the previous reports.

In summary, this study attempted to uncover the effects of deletions of four gene clusters encoding antibiotic substances on γ‐PGA synthesis. Their effects on swarming and biofilm formation in *B. amyloliquefaciens* LL3 were also studied. The γ‐PGA yield of LL‐IS (Δ*itu*Δ*srf*) (4.5 g/L) increased by 36.4% compared with the wild‐type strain (3.3 g/L), and the culture viscosity decreased by 24.6%, which is favorable for industrial production.

## Funding Information

This work was supported by the National Key Basic Research Program of China (“973″ ‐Program) (2012CB725204), the National Key Technology Support Program (2015BAD16B04), the Natural Science Foundation of China (grant nos. 31470213 and 31170030), and the Project of Tianjin, China (13JCZDJC27800, 14ZCZDSF00009, and 15ZCZDNC00450).

## Conflict of Interest

None declared.
