# Supplementary material for: Mutations in genes encoding antibiotic substances increase the synthesis of poly‐γ‐glutamic acid in Bacillus amyloliquefaciens LL3
Source: Microbiologyopen. 2016 Aug 18;6(1):e00398. doi: 10.1002/mbo3.398 (PMC5300885; doi:10.1002/mbo3.398)
Supplement: Supplementary file 1 [file MBO3-6-0-s001.doc]

**
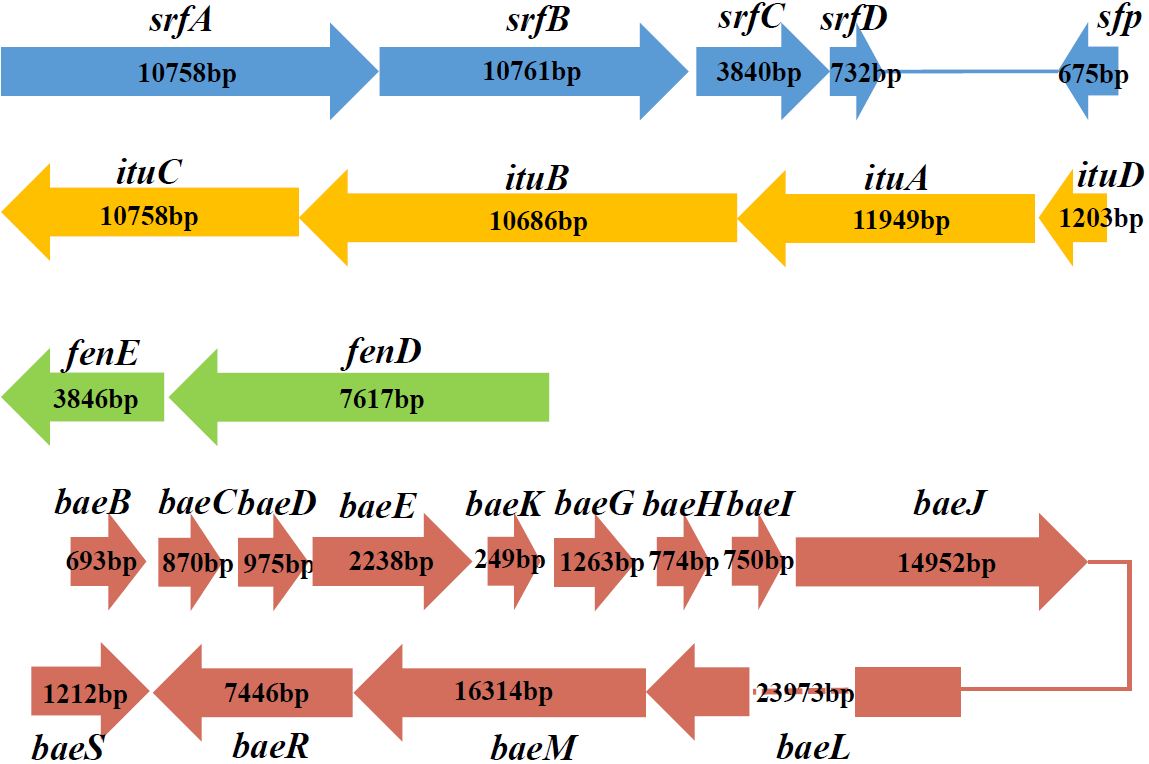
**

**Fig. S1** The four gene clusters of surfactin, iturin A, fengycin and bacillaene (polyketone) in *B. amyloliquefaciens* LL3.


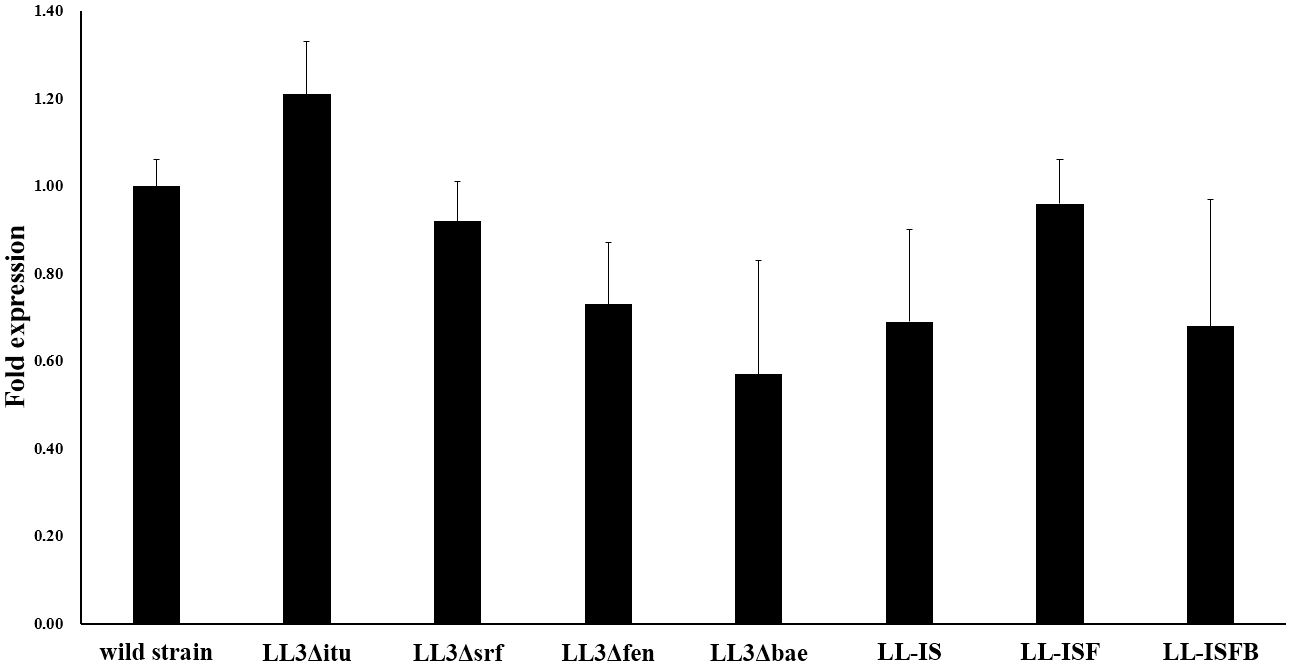


**Fig. S2** The qRT-PCR results of *pgsB* gene in the wild-type strain and the mutant strains.


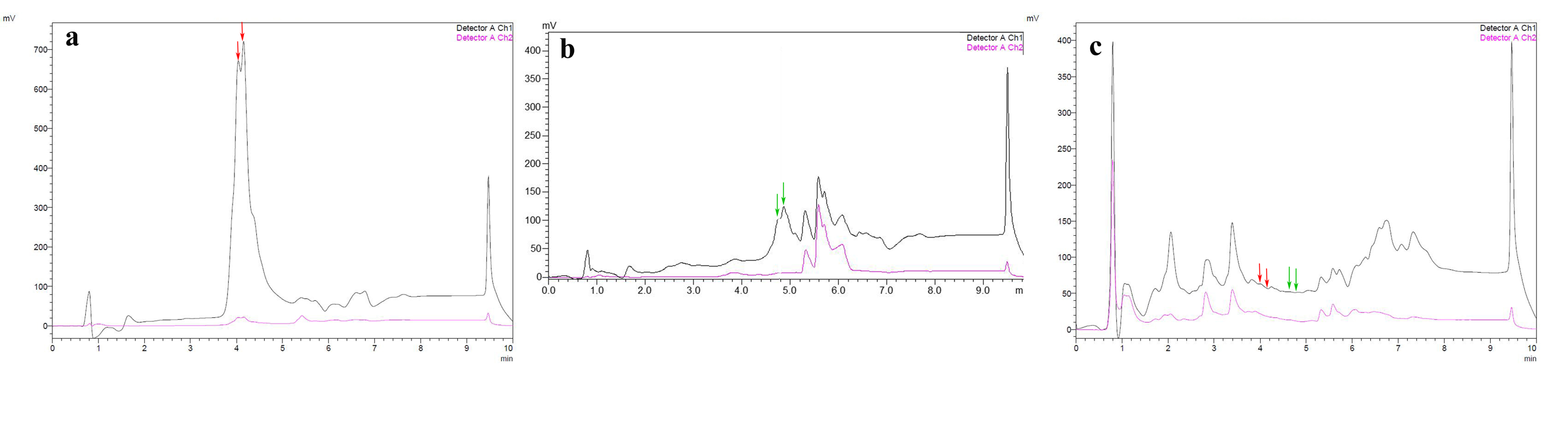


**Fig. S3** HPLC spectrograms of fengycin **(a)**, iturin A **(b)** and LL3Δ*upp* culture **(c)**. The arrows in red and green point to the characteristic peaks of fengycin and iturin A, respectively.
